# Randomised phase II trial of weekly ixabepilone ± biweekly bevacizumab for platinum-resistant or refractory ovarian/fallopian tube/primary peritoneal cancer

**DOI:** 10.1038/s41416-022-01717-6

**Published:** 2022-02-11

**Authors:** Dana M. Roque, Eric R. Siegel, Natalia Buza, Stefania Bellone, Dan-Arin Silasi, Gloria S. Huang, Vaagn Andikyan, Mitchell Clark, Masoud Azodi, Peter E. Schwartz, Gautam G. Rao, Jocelyn C. Reader, Pei Hui, Joan R. Tymon-Rosario, Justin Harold, Dennis Mauricio, Burak Zeybek, Gulden Menderes, Gary Altwerger, Elena Ratner, Alessandro D. Santin

**Affiliations:** 1 Marlene and Stewart Greenebaum Comprehensive Cancer Center, University of Maryland School of Medicine, Baltimore, MD USA; 2 University of Arkansas for Medical Sciences, Little Rock, AR USA; 3 Smilow Comprehensive Cancer Center, Yale School of Medicine, New Haven, CT USA; 4 Division of Gynecologic Oncology, Mercy Clinic, St. Louis, MO USA

**Keywords:** Translational research, Phase II trials

## Abstract

**Background:**

This multi-center RP2 study assessed activity/safety of ixabepilone + bevacizumab compared to ixabepilone in platinum-resistant/refractory ovarian/fallopian tube/primary peritoneal cancer. Additional objectives were to examine the role of prior bevacizumab and taxanes, and explore class III-ß-tubulin (TUBB3) as a predictive biomarker.

**Methods:**

Participants were randomised to receive ixabepilone 20 mg/m^2^ days 1, 8, 15 with (IXA + BEV) or without (IXA) bevacizumab 10 mg/kg days 1, 15 every 28 days. Patients were stratified by prior BEV. The primary endpoint was PFS. OS, safety, and ORR served as secondary endpoints.

**Results:**

Among 76 evaluable patients who received IXA + BEV (*n* = 39) compared to IXA (*n* = 37), the ORR was 33% (*n* = 13) versus 8% (*n* = 3)(*P* = 0.004), durable at 6 months in 37% (*n* = 14) and 3% (*n* = 1) (*P* < 0.001). BEV significantly improved PFS (median:5.5 vs 2.2 months, HR = 0.33, 95%CI 0.19–0.55, *P* < 0.001) and OS (median:10.0 vs 6.0 months, HR = 0.52, 95%CI 0.31–0.87, *P* = 0.006). Both regimens were well-tolerated. TUBB3 expression did not predict response. Subgroup analyses revealed minimal effect of prior BEV or taxane resistant/refractory status on response to IXA + BEV.

**Conclusions:**

IXA + BEV is a well-tolerated, effective combination for platinum/taxane-resistant ovarian cancer that extends PFS and likely OS relative to IXA monotherapy. Prior receipt of BEV should not preclude the use of IXA + BEV. TUBB3 is not a predictive biomarker.

**Clinical trial registration:**

NCT3093155.

## Introduction

Ovarian cancer patients suffer from the poorest survival rates among those with gynaecologic malignancies [1]. Globally, there are an estimated 296,414 cases and 184,799 deaths annually from ovarian cancer [2]. While initial treatment most often leads to remission, progressive chemoresistance with each recurrence eventually leads to death [3]. Few effective combinations for heavily pre-treated patients currently exist.

The AURELIA trial demonstrated that the addition of bevacizumab (BEV), a recombinant humanised monoclonal antibody against vascular endothelial growth factor (VEGF), to cytotoxic chemotherapy (pegylated liposomal doxorubicin, topotecan, or weekly paclitaxel) significantly improved progression-free survival (PFS) by 3 months and boosted response rates by 15% in platinum-resistant patients with ≤2 lines of prior treatment. Unfortunately, this approach offered no overall survival (OS) benefit [4] and has been criticised as cost-ineffective [5].

Epothilones are microtubule-stabilising agents that induce mitotic arrest, abrogate microtubule dynamics [6], and affect microtubule-dependent intracellular transport [7], leading to cell death. Ixabepilone (IXA; Ixempra® R-Pharm, NJ) is a semi-synthetic second-generation analogue of epothilone B with activity in pre-treated ovarian [8], breast [9, 10], pancreatic [11, 12], lung [13], and prostate [14, 15] cancers. It is currently FDA-approved in locally advanced or metastatic breast cancer [16]. In GOG 126M, a phase II evaluation of IXA 20 mg/m^2^ days 1, 8, 15 every 28 days in platinum/taxane-resistant ovarian cancer, objective response (OR) rate was 14.3% with a 4.4 month time to progression and OS of 14.8 months [8]. Subsequent to this, we reported the encouraging activity of IXA + BEV in a retrospective analysis of ovarian cancer patients [17].

Epothilones may retain activity in taxane-treated patients since they tend not to be substrates for p-glycoprotein drug-exportation pumps and they preserve affinity to the binding domain despite tumoral upregulation of class III ß tubulin (TUBB3) in place of the constitutively expressed class I isotype [18, 19]. Preclinical and retrospective studies in ovarian cancer have suggested TUBB3 may serve as a marker of resistance to paclitaxel and sensitivity to epothilones [20–22]; however, clinical data remain inconsistent [13, 23, 24].

Herein, we conducted a phase II prospective multi-site comparison of ixabepilone with bevacizumab (IXA + BEV) versus ixabepilone monotherapy (IXA) to (1) assess efficacy and safety of the combination, (2) explore TUBB3 expression by immunohistochemistry as a predictive biomarker for response, and (3) describe differences in response to the regimen in relationship to previous treatment with BEV and taxanes.

## Methods

### Study design and conduct

**Fig. 1:**
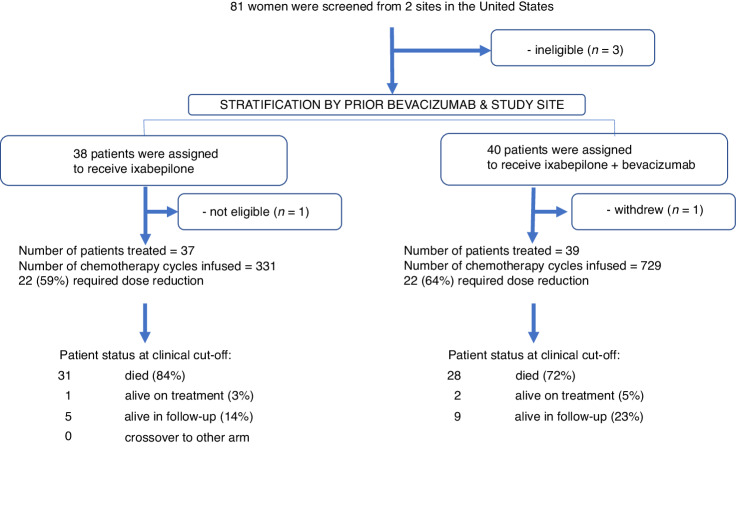
Consolidated Standards of Reporting Trials (CONSORT) diagram shows progress through randomization.

This was an investigator-initiated phase II randomised open-label trial (NCT03093155) conducted at Smilow Cancer Hospital at Yale University and the Greenebaum Comprehensive Cancer Center at the University of Maryland. This investigation was conducted in accordance with the Declaration of Helsinki and approved by local Human Investigations Committees. All patients provided written informed consent to participate in this study. The primary objective was to compare IXA monotherapy at 20 mg/m^2^ intravenously days 1, 8, and 15 of a 28-day cycle alone or with BEV 10 mg/kg intravenously days 1 and 15 administered until disease progression, death, or prohibitive toxicity (Fig. 1) for an increase in progression-free survival (PFS). Study participants were stratified by study site and previous receipt of BEV with a 1:1 allocation using a dynamic randomisation procedure to minimise stratification-factor imbalance between arms. There were no significant amendments made during its conduct, though enrollment was briefly suspended during the COVID-19 pandemic. Power calculations assumed a median PFS for IXA monotherapy to be 5 months, given observations from GOG 126 M [8]. Based on the AURELIA trial [4] and our own retrospective data [17], we anticipated that the addition of BEV to IXA would double median PFS. Assuming an accrual of 5 participants/month, the trial was designed to recruit 88 participants over 17.6 months to reach 80 participants, accounting for a 10% drop-out rate. We required 80% power at 5% *α* to detect a 2-fold increase in PFS via one-sided log-rank test while allowing for a single interim analysis for efficacy and futility. Calculations conducted in East v 6.4 (Cytel, Inc, Cambridge, MA) using the null variance estimator along with the O’Brien-Fleming spending functions for both alpha and beta required 28 PFS events in the interim and 56 PFS events in the final analysis. The first participant enrolled in March 2017. Because the COVID-19 pandemic slowed recruitment, we terminated enrollment after 78 participants and the occurrence of 61 PFS events. Final efficacy analyses were conducted in November 2020. The full protocol is provided in the Supplemental Appendix. Similar to previously published definitions by others [25], patients were considered *taxane-resistant* if they demonstrated disease progression within 6 months of paclitaxel/docetaxel administration. Patients were considered *taxane-refractory* if they progressed while receiving a taxane or demonstrated persistence of disease on end-of-treatment assessment that prompted initiation of a new line of therapy.

### Eligibility

All participants were ≥18 years and had platinum-resistant (i.e. platinum-free interval <6 months) or refractory (i.e. disease progression during or ≤4 weeks after last dose of platinum) histologically confirmed epithelial (non-mucinous) ovarian, fallopian tube, or primary peritoneal carcinoma. All participants had measurable disease per RECIST (Response Evaluation Criteria in Solid Tumors) v1.1 [26]. Participants had to exhibit a performance status of 0–2 [27]. Any prior debulking status was permitted. Participants must have received prior treatment with paclitaxel either 3-weekly or weekly (≥3 cycles). There was no limit on prior lines including BEV therapy.

### Immunohistochemistry

Tumour specimen was required. Recurrent tumour was preferred, though archival pre-treatment specimens were also permitted. Sections were cut at 4 μm and stained with class III beta-tubulin monoclonal antibody (TUJ1, Covance, Berkeley, CA) at 1:500 dilution. Staining intensity (cytoplasmic and membranous) was assessed using a semi-quantitative scoring system: 0, negative (none); 1+, focal, weak; 2+, diffuse weak or focal moderate; 3+, diffuse moderate or focal strong; 4+, diffuse, strong immunoreactivity [21, 22, 28]. All slides were reviewed by surgical pathologists (NB, PH) to derive a consensus score. Pathologists were blinded to outcomes during IHC scoring.

### Study drugs

In certain settings, weekly paclitaxel may improve efficacy compared to 3-weekly dosing [29, 30]. IXA has also been studied using both 3-weekly and weekly dosing schemes [31]. We chose weekly dosing for consistency with previous studies by the GOG (126M) [8] and others [32] suggesting this might minimise haematologic and neuropathic toxicity. IXA was provided by RPharm-US LLC, Princeton, NJ. BEV was supplied commercially. Biosimilars were not permitted.

### Endpoints

Computed tomography was performed every 2 cycles. The primary endpoint was PFS, defined as time from randomisation to progression or death. Secondary endpoints were OS, defined as time from randomisation to death from any cause, and safety as defined by Common Terminology Criteria for Adverse Events (CTCAE) v.4 [33]. Best response was based on RECIST v1.1. OR consisted of complete response (CR) or partial response (PR), and it did not have a durability requirement. Durable Disease Control (DDC) was defined as CR, PR, or stable disease (SD) ≥6 months from date of best response.

### Statistical analyses

Fisher’s exact and Wilcoxon rank-sum tests at *α* = 0.05 were used to assess differences between treatment arms in TUBB3 immunohistochemistry and adverse events (AEs) or serious adverse events (SAEs). PFS and OS were analysed using the Kaplan–Meier method with one-sided log-rank tests and Cox regression. One-sided chi-square tests were employed for comparison of OR, DDC, and taxane resistant/refractory status. Subgroup analyses were performed and illustrated by Forest plots. The datasets generated and/or analysed during the current study are available from the corresponding author on reasonable request.

## Results

### Participants

Eighty-one participants were screened, and 78 were randomised (Fig. 1). One withdrew consent and one was found ineligible, leaving 76 evaluable for efficacy. Patient and disease characteristics are provided in Table 1. There was no evidence of imbalance. Notably, 49% of participants (51% in the IXA arm and 46% in the IXA + BEV arm) had received >3 prior lines of chemotherapy (p-0.82). Fourteen (18.4%) participants (11% in the IXA arm versus 26% with IXA + BEV) had platinum-refractory disease (*p* = 0.14). Within the IXA arm, 9 (24%) of patients were taxane-resistant, 10 (27%) were taxane-refractory; within the IXA + BEV arm, 13 (33%) were taxane-resistant, 13 (33%) were taxane-refractory (*p* = 0.44). Prior treatment included dose-dense/weekly paclitaxel in 27% (*N* = 10) and 23% (*N* = 9) within the monotherapy and combination therapy arms, respectively. Among all patients, median time from last taxane was 399 days (384 for the IXA arm and 413 for the IXA + BEV arm). Roughly 46% (17/37) in the IXA arm and 67% (26/39) in the IXA + BEV arm had progressed after at least one AURELIA regimen. Approximately 38% (14/37) in the IXA arm and 10/39 (26%) in the IXA + BEV arm had failed PARP inhibition. At the time of analysis, 3 participants remained on treatment with SD, with one deemed durable >6 months.

**Table 1:** Patient characteristics.

	IXA (*n* = 37)	IXA + BEV (*n* = 39)
Age in years, median (range)	67 (50-88)	67 (40-78)
Race, % (*N*)		
White	73% (27)	80% (31)
Black	22% (8)	10% (4)
Other	5% (2)	10%(4)
Ethnicity, % (*N*)		
Hispanic	8% (3)	3% (1)
Non-Hispanic	92% (34)	95% (37)
Unknown	0	3% (1)
Histology, % (*N*)		
Serous	78% (29)	87% (34)
Carcinosarcoma	6% (2)	3% (1)
Other	16% (6)	10% (4)
ECOG Performance Status, % (*N*)		
0–1	84% (31)	92% (36)
2	16% (6)	8 % (3)
Prior Lines of Chemotherapy, % (*N*)		
≤3	49% (18)	54% (21)
>3	51% (19)	46% (18)
Prior PARP inhibitor		
Yes	38% (14)	26% (10)
No	62% (23)	74% (29)
Prior Bevacizumab, % (*N*)		
Yes	57% (21)	54% (21)
No	43% (16)	46% (18)
Prior weekly Paclitaxel (for first line treatment or treatment of recurrence), % (*N*)		
Yes	27% (10)	23% (9)
No	73% (27)	77% (30)
Prior receipt of an AURELIA regimen, % (*N*)		
With Bevacizumab	16% (6)	21% (8)
weekly paclitaxel	17% (1)	50% (4)
pegylated liposomal doxorubicin	33% (2)	50% (4)
topotecan	17% (1)	0
>AURELIA regimen	33% (2)	0
Without Bevacizumab	30% (11)	46% (18)
weekly paclitaxel	9% (1)	11% (2)
pegylated liposomal doxorubicin	64% (7)	67% (12)
topotecan	9% (1)	17% (3)
>1 AURELIA regimen	18% (2)	5% (1)
Platinum refractory/resistant disease, % (*N*)		
Refractory	11% (4)	26% (10)
Resistant	89% (33)	74% (29)
Taxane refractory/resistant disease, % (*N*)		
Refractory	27% (10)	33% (13)
Resistant	24% (9)	33% (13)
Exposed	49% (18)	33% (13)

*ECOG* Eastern Cooperative Oncology Group.

The study arms were well-balanced across all variables examined with no significant differences between the two groups.

### Treatment

Individual follow-up intervals had a median (range) of 8.5 (0.59–40.1) months. Total time on study was 829.5 months. A total of 409 cycles were administered to 76 patients to yield a median of 4 (interquartile range: 2–6/patient, range:1–40). Mean (±standard deviation) cycles received among the IXA versus IXA + bev cohorts was 3.4 ± 2.1 and 7.3 ± 7.7. Dose reductions were required by 64% of participants treated with IXA + BEV arm and 59% treated with IXA. Main dose-limiting toxicities were peripheral neuropathy (39 events), neutropenia (18 events), and fatigue (129 events).

### Primary endpoint: PFS

**Fig. 2:**
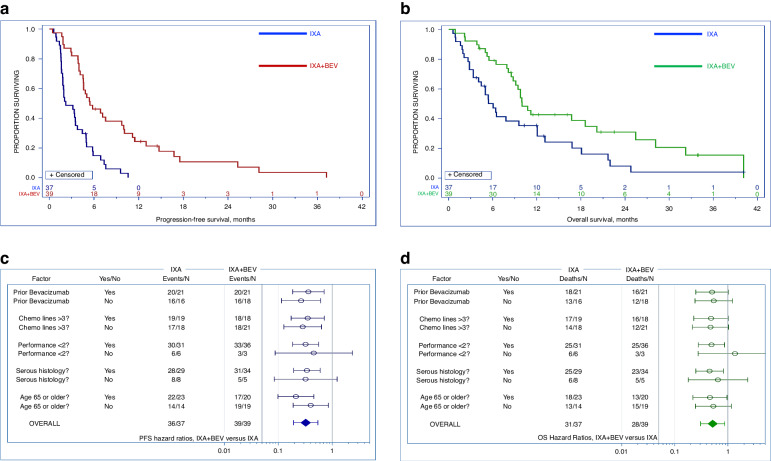
Progression-free survival, overall survival, and subgroup analyses. **a** Progression-free survival: use of bevacizumab (BEV) with ixabepilone (IXA) significantly extended progression-free survival (5.5 vs 2.2 months, HR 0.33, 95% CI 0.19–0.55, *p* < 0.001) compared to IXA alone. **b** Overall Survival: overall survival was significantly longer in patients who received BEV in conjunction wiht IXA (10.0 vs 6.0 months, HR 0.52, 95% CI 0.31–0.87, *p* < 0.006). **c** Hazard ratios for progression-free survival versus treatment arm by subgroup: progression-free survival hazard radios were similar between arms among patients with prior BEV exposure (HR 0.36, 95% CI: 0.19–0.72, *p* = 0.003) and those who were BEV-naive (HR 0.27, 95%CI: 0.12–0.62, *p* = 0.002). Stratification by pre-treatment status, age, histology, and performance status are also shown. Error bars denote 95% confidence intervals (CI). **d** Hazard ratios for overall survival versus treatment arm by subgroup: similar hazard ratios for overall survival were observed between arms among patients with prior BEV exposure (HR 0.50, 95%: 0.25–1.02, *p* = 0.058) and those who were BEV-naive (HR 0.54, 95% CI: 0.24–1.22, *p* = 0.14). Stratification by pre-treatment status, age, histology, and performance status are also shown. Error bars denote 95% CI.

There were 72 PFS events (58 progressions and 14 deaths without progression). Use of IXA + BEV significantly extended median PFS (5.5 vs 2.2 months, HR 0.33, 95% CI 0.19–0.55, *p* < 0.001) compared to IXA alone (Fig. 2a). Resistance or refractoriness to taxanes did not influence PFS between treatment arms. Two-factor Cox regression with taxane resistant/refractory status and treatment arm as binary factors showed that taxane status had little modulatory effect on treatment efficacy (interaction *p* = 0.64) and little direct effect on PFS (main-effect *p* = 0.82). Comparing treatment arms while controlling for the effect of taxane resistance/refractoriness yielded an adjusted HR 0.33, 95% CI 0.20–0.56).

### Secondary endpoints: OS, OR rate (ORR), safety

There were 59 deaths (Fig. 1). OS was also significantly longer in participants who received IXA + BEV compared to IXA monotherapy (10.0 vs 6.0 months, HR 0.52, 95% CI 0.31–0.87, *p* = 0.006) (Fig. 2b). Resistance or refractoriness to taxanes did not influence OS between treatment arms. Two-factor Cox regression with taxane resistant/refractory status and treatment arm as binary factors showed that taxane status had little modulatory effect on treatment efficacy (interaction *p* = 0.68) and little direct effect on OS (main-effect *p* = 0.44). Comparing treatment arms while controlling for the effect of taxane resistance/refractoriness yielded an adjusted HR of 0.55, 95% CI 0.32–0.94). Participants in the IXA + BEV arm had a higher ORR (33% versus 8%, *p* = 0.004). Taxane resistance/refractoriness had no independent effect on ORR (odds ratio 1.36, 95% CI 0.40–4.69). There were no CRs. SD occurred in 54% (*n* = 21) and 51% (*n* = 19), respectively. There were 74 participants evaluable for DDC, which was achieved in 37% (14) in the IXA + BEV arm and 3% (*n* = 1) of the IXA arm (*p* = 0.0001). No patient crossed over from the IXA arm to receive IXA + BEV. Among patients with prior exposure to weekly paclitaxel, there was only one partial response, and this occurred in the IXA + BEV group. The disease stabilisation rate was approximately 50% in both arms; the remainder of patients exhibited progressive disease.

There were no new safety signals. Seventy participants reported 681 AEs possibly, probably, or definitely related to study drug. A total of 26 SAEs related to study drug were reported among 14 participants (IXA: *n* = 6, 43%; IXA + BEV: *n* = 8, 57%) (*p* = 0.77) (Table 2). There was a single bowel perforation in the IXA + BEV arm. There were no particular predisposing factors identified in her treatment history, which consisted of neoadjuvant carboplatin/paclitaxel followed by interval debulking, carboplatin/pegylated liposomal doxorubicin, interval debulking, carboplatin/pegylated liposomal doxorubicin, anastrazole, and topotecan. Hypertension was more common in participants who received IXA + BEV (*n* = 14, 36%) compared to IXA (*n* = 3, 8%) (*p* = 0.005); most cases were grade 1–2. Participants in this group were also more likely to report sensory neuropathy (*n* = 20, 51% versus *n* = 7, 19%, *p* = 0.004) and musculoskeletal (*n* = 22, 56% versus *n* = 9, 24%, *p* = 0.006) AEs. Grade 4/5 events were evenly distributed. Six participants experienced a grade 4 event (sepsis, *n* = 2 [IXA, IXA + BEV]; neutropenia, *n* = 2 [IXA, IXA + BEV]; pneumonia, *n* = 1 [IXA]; anaemia, *n* = 1 [IXA + BEV]). Two participants experienced a grade 5 adverse event (cardiac tamponade, *n* = 1 [IXA]; renal failure, *n* = 1 [IXA + BEV]), both of which were deemed unlikely to be related to treatment. Distribution and grading of AEs has been provided in the Supplemental Appendix.

**Table 2:** Serious adverse events.

Category	Arm	Grade								
		1		2		3		4		SAE
		SAEs	Patients	SAEs	Patients	SAEs	Patients	SAEs	Patients	Total
Blood and lymphatic system disorders										
IXA		▪	▪	1	1	1	1	▪	▪	2
IXA+BEV		▪	▪	1	1	▪	▪	▪	▪	1
Ear and labyrinth disorders										
IXA		▪	▪	▪	▪	▪	▪	▪	▪	0
IXA+BEV		▪	▪	▪	▪	1	1	▪	▪	1
Gastrointestinal disorders										
IXA		▪	▪	1	1	1	1	▪	▪	2
IXA+BEV		▪	▪	3	2	6	6	▪	▪	9
General disorders and administration site conditions										
IXA		▪	▪	▪	▪	1	1	▪	▪	1
IXA+BEV		▪	▪	▪	▪	▪	▪	▪	▪	0
Infections and infestations										
IXA		▪	▪	▪	▪	▪	▪	1	1	1
IXA+BEV		▪	▪	▪	▪	▪	▪	▪	▪	0
Investigations										
IXA		1	1	▪	▪	▪	▪	1	1	1
IXA+BEV		▪	▪	▪	▪	▪	▪	▪	▪	0
Metabolism and nutrition disorders										
IXA		1	1	▪	▪	2	2	▪	▪	3
IXA+BEV		▪	▪	▪	▪	1	1	▪	▪	1
Nervous system disorders										
IXA		▪	▪	1	1	▪	▪	▪	▪	1
IXA+BEV		▪	▪	▪	▪	▪	▪	▪	▪	0
Renal and urinary disorders										
IXA		▪	▪	▪	▪	▪	▪	▪	▪	0
IXA+BEV		▪	▪	▪	▪	1	1	▪	▪	1
Respiratory, thoracic and mediastinal disorders										
IXA		▪	▪	1	1	▪	▪	▪	▪	1
IXA+BEV		▪	▪	▪	▪	▪	▪	▪	▪	0
Total										
IXA		2	2	3	3	5	5	2	2	12
IXA+BEV		▪	▪	5	4	9	9	▪	▪	14
ALL		2	2	8	7	14	14	2	2	26

A total of 26 SAEs related to study drug were reported among 14 patients and the number of patients affected did not differ between arms (*p* = 0.77).

### Subgroup analyses

A pre-specified analysis of stratification by receipt of prior BEV minimally affected hazard ratios for PFS and OS between arms (Fig. 2c, d). Among participants who received combination therapy, median PFS in BEV-naive individuals was 9.9 months (95% CI: 5.5–14.7) versus 4.6 months (95% CI: 4.0–6.9) in those had a history of prior BEV therapy (*p* = 0.058); median OS was 18.6 months (95% CI: 9.8–25.4) versus 9.4 months (95% CI: 6.4–16.7), respectively (*p* = 0.21). Post-hoc stratification by other potential contributors to outcome including heavy pre-treatment, performance status, age, and histology failed to drive large effects.

### Correlative studies

No significant differences in TUBB3 expression were apparent between response groups when examined by best RECIST or DDC, even upon controlling for treatment arm. Immunohistochemistry could not be performed for 18% (*n* = 14). No TUBB3 staining was observed in 13% (*n* = 10); when detectable, staining was 1+ in 11% (*n* = 8), 2+ in 28% (*n* = 21), 3+ in 24% (*n* = 18), and 4+ in 7% (*n* = 5). Representative immunohistochemistry images are included in the Supplemental Appendix.

## Discussion

In this prospective, randomised phase II study, the addition of bevacizumab to ixabepilone resulted in statistically significant improvement in PFS and suggested an OS benefit in platinum/taxane-resistant/refractory ovarian cancer. The addition of BEV to chemotherapy has previously failed to show improvement in OS spanning the upfront setting (GOG 218, ICON-7) [34, 35], platinum-sensitive recurrent setting (OCEANS, GOG-213) [36, 37], and platinum-resistant recurrent setting (AURELIA) [4]. Of note, neither this study nor the AURELIA study were powered to demonstrate OS benefit; interpretation of the AURELIA trial may have been further conflated by a cross-over rate of 40% [38].

The response rate observed for IXA monotherapy in our study (8%) is lower than that described previously in recurrent ovarian cancer for not only IXA [8] and epothilone B (patupilone) [39], but also for other single-agent chemotherapies (12–16%) [4]. This may be due to inclusion of platinum-refractory participants (18.4%) with heavy pre-treatment, as previous studies of IXA restricted participants to only one prior line following platinum-taxane chemotherapy, or dose reduction. Response rates for IXA as a single-agent range from 6%-21% [40] for platinum-resistant non-small cell lung [41], renal cell [42], and urothelial carcinoma [43]. The response rate in breast cancer previously treated with taxanes is 22% [44].

A dose reduction by 20% was necessary in 59% (IXA) and 64% (IXA-BEV) of participants. This enabled continuation of IXA after recovery in all. These results suggest that a weekly dose of 16 mg/m^2^ may represent an effective, better tolerated starting dose for heavily pretreated patients.

Interestingly, we found the response rate for IXA + BEV to compare favourably to that of BEV in combination with other chemotherapy regimens as observed in the AURELIA trial (33% versus 27%, respectively) (Table 3). Subgroup analyses AURELIA showed ORR in the paclitaxel cohort to be improved by 23.1% by the addition of BEV to paclitaxel (53.3% versus 30.2%), similar to the improvement we observed by the addition of BEV to IXA (25%). This is in contrast to improvements of 17% in the topotecan cohort (17% versus 0%) and 5.9% (13.7% versus 7.8%) in the pegylated liposomal doxorubicin cohort [45]. Because AURELIA did not randomise chemotherapy cohorts, no firm conclusions can be drawn. Notably, AURELIA did not report specifically on paclitaxel resistant/refractory status and excluded platinum-refractory patients. The performance of paclitaxel with BEV in a more heavily pre-treated population is uncertain, as existing reports are largely retrospective [46]. In this study, >50% of patients were taxane-resistant or refractory, and this did not affect PFS, OS, or ORR. Our definitions may have underestimated the number of patients with biologic evidence of paclitaxel resistance due to the prescription of other doublets (i.e. carboplatin/pegylated liposomal doxorubicin or carboplatin/gemcitabine) at the time of platinum-sensitive recurrence and failure to re-challenge with carboplatin/paclitaxel. Previous reports by the Gynecologic Oncology Group have illustrated the importance of paclitaxel dose intensity on response [47]; in patients with resistance to 3-weekly paclitaxel dosing, up to 21% may still respond to weekly administration possibly due to a more complete exploitation of cell-cycle specificity or direct effects on angiogenesis, among other hypotheses. In this cohort, roughly a quarter of patients in both arms had already received weekly paclitaxel, and disease stabilisation was achieved in approximately 50% across both arms. In the present study, 46% of patients in the IXA arm and 67% in the IXA + BEV arm had also progressed after at least one AURELIA regimen, thus, our data suggest that IXA + BEV may retain activity following an AURELIA regimen. As with the AURELIA trial, the present study lacked a BEV monotherapy control arm. In previous studies of bevacizumab monotherapy [48–50] only partial responses were achieved, with overall response rates of 10.1% [48] to 21% [49]. In GOG-170D, the ORR to single-agent BEV was 21% with a median PFS of 4.7 months, though only 58% of participants were platinum-resistant and 66% had received 2 prior regimens [49]. In a study restricted to platinum-resistant patients, patients received at least 2 but no more than 3 prior regimens with an ORR of 15.9% [50].

**Table 3:** Comparison of ixabepilone/bevacizumab (BEV) with chemotherapy/(BEV) in the AURELIA trial.

	AURELIA	Present study
Percent platinum-refractory	0%	18%
Pre-treatment status	(i.e. >3 prior lines) 0% (chemotherapy) 0% (chemotherapy + BEV)	(i.e. >3 prior lines) 51% (IXA) 46% (IXA + BEV)
Prior anti-angiogenic therapy	8% (chemotherapy) 7% (chemotherapy and BEV)	57% (IXA) 54% (IXA + BEV)
Response rate (RECIST)	12% (chemotherapy) 27% (chemotherapy + BEV)	8% (IXA) 33% (IXA + BEV)
weekly paclitaxel^a^	30.2% 53.3% (+BEV)	N/A
topotecan*	0% 17% (+BEV)	N/A
pegylated liposomal doxorubicin^a^	7.8% 13.7% (+BEV)	N/A
PFS (months)		
Chemotherapy	3.4 (95% CI 2.2–3.7)	N/A
Chemotherapy + BEV	6.7 (95CI 5.7–7.9)	N/A
IXA	N/A	2.2 (95% CI 1.8–3.8)
IXA + BEV	N/A	5.5 (95% CI 4.6–10.0)
Weekly Paclitaxel^a^	3.9 (95% CI 3.5–5.6)	N/A
Weekly Paclitaxel + BEV^a^	10.4 (95% CI 7.9–11.9)	N/A
IXA + BEV (BEV-naive)	N/A	9.9 (95% CI 5.5–17.5)
IXA + BEV (prior BEV)	N/A	4.6 (95% CI 4.0–6.9)
OS (months)		
Chemotherapy	13.3 (95% CI 11.9–16.4)	N/A
Chemotherapy + BEV	16.6 (95% CI13.7–19.0)	N/A
IXA	N/A	6.0 (95% CI 4.1–12.1)
IXA + BEV	N/A	10.0 (95% CI 9.1–20.2)
Weekly Paclitaxel^a^	13.2 (95% CI 8.2–19.7)	N/A
Weekly Paclitaxel + BEV^a^	22.4 (95% CI 16.7–26.7)	N/A
IXA + BEV (BEV-naive)	N/A	18.6 (95% CI 9.8–25.4)
IXA + BEV (prior BEV)	N/A	9.4 (95% CI 6.4–16.7)
Bowel perforation with BEV	2% (*n* = 4)	2.5% (*n* = 1)

*CI* confidence interval.

^a^Ancillary analyses by Poveda et al. (2015) [45].

The effects we observed with IXA + BEV are consistent with in vitro observations that sublethal concentrations of microtubule-stabilising agents inhibit angiogenesis [51, 52], a feature generally not observed with platinum or anthracyclines [53]. This may be modulated by disturbances of actin stress-fibre formation affecting cell polarity and lamellipodia. Microtubule-stabilising agents may down-regulate VEGF [54]. In human xenografts, activity is noted for IXA + BEV even when resistance exists to the individual agents [51]. The combination of IXA + BEV results in significantly more inhibition of endothelial cell invasion and proliferation than paclitaxel [51]. Endothelial cells treated with IXA + BEV demonstrate 75% reduction in tumour vessel density, compared to 15% with paclitaxel+BEV [51]. Normalisation of tumour vasculature leading to better drug delivery may contribute [55].

One bowel perforation occurred in our study [56–58]. While more patients experienced peripheral neurologic and musculoskeletal toxicity in the IXA + BEV arm, we did not control for cumulative prior toxicities. Due to prolonged clinical benefit, the IXA-BEV group received more than double the number of infusions (Fig. 1). Sensory neuropathy was also greater in the chemotherapy+BEV arm of AURELIA [4]; some [59] have suggested a class effect in which VEGF inhibition hinders the regeneration of neurons.

In the present study, the addition of BEV to IXA benefitted women regardless of prior BEV treatment. Mechanisms that may contribute to BEV resistance include activation of alternative angiogenesis pathways through hypoxia-mediated pathways [60–64] and suppression of oxidative phosphorylation [65]. Nevertheless, a number of retrospective [66, 67] and prospective studies [37, 68, 69] have illustrated the benefit and safety of re-treatment with BEV after prior BEV in ovarian cancer.

Financial modelling of the AURELIA trial asserted that the price (2015) of bevacizumab must be reduced by 20% to be cost-effective [5]. The first biosimilar (bevacizumab-awwb, Amgen-Allergan) entered the market in 2019 at 12% below the price of bevacizumab [70], and may facilitate employment of IXA and a biologic in a cost-effective fashion.

Predictive biomarker discovery likewise has enormous potential for treatment allocation and healthcare cost containment. In this study, TUBB3 was not predictive of response to IXA. This may reflect sampling error in the context of intra-tumoral heterogeneity, as only a single specimen representing either primary or metastatic disease was used to define TUBB3 profile, and incomplete immunohistochemistry in 18% [71]. Most specimens represented chemotherapy-naive tumour and results might have been confounded by other mechanisms that control TUBB3 expression, such as hypoxia via transcription factors HIF-1α/HIF-2α [72, 73] epigenetic modifications [74] or differences in warm ischaemia time and treatments rendered. We have previously shown that neoadjuvant chemotherapy results in decreased expression of class III β-tubulin in ovarian cancer stroma, thought to represent normalisation of the tumour microenvironment in response to treatment [21]. Microtubule-associated proteins (MAPs) dictate dynamic instability of the cellular cytoskeleton and represent promising biomarkers of resistance to microtubule-stabilising agents, but remain under-studied in prospective settings [75]. MAPs such as tau have been associated with reduced paclitaxel binding [76, 77]. The plus-end-binding protein, EB1, correlates with paclitaxel resistance in breast cancer [78]. Microtubule-destabilising MAPs such as stathmin attenuate paclitaxel binding and predict poor outcome in ovarian cancer patients treated with carboplatin/paclitaxel [79]. IXA and paclitaxel exert differential effects on MAPs possibly due to drug-specific conformational changes in the carboxy terminus of microtubules, therefore MAPs are relevant biomarkers to explore in relationship to therapeutic response to these agents [80].

In summary, the combination of weekly ixabepilone and biweekly bevacizumab represents a well-tolerated regimen with encouraging activity, which should be considered in the armament of agents available for platinum- and taxane-resistant disease even in the heavily pre-treated setting not fully captured by the AURELIA trial. The addition of BEV to IXA significantly improves PFS compared to IXA monotherapy, with suggestion of OS benefit. Prior receipt of BEV should not preclude use of this combination. TUBB3 protein expression by immunohistochemistry does not predict response to ixabepilone. Future studies should focus on refinement of predictive biomarkers, as well as potential application of this combination to other gynecologic primaries such as platinum- and taxane-resistant endometrial cancer.

### Supplementary information

SUPPLEMENTAL APPENDIX

## Data Availability

The datasets generated and/or analysed during the current study are available from the corresponding author on reasonable request.
