# Correction: Randomised phase II trial of weekly ixabepilone ± biweekly bevacizumab for platinum-resistant or refractory ovarian/fallopian tube/primary peritoneal cancer

**DOI:** 10.1038/s41416-024-02628-4

**Published:** 2024-03-04

**Authors:** Dana M. Roque, Eric R. Siegel, Natalia Buza, Stefania Bellone, Dan-Arin Silasi, Gloria S. Huang, Vaagn Andikyan, Mitchell Clark, Masoud Azodi, Peter E. Schwartz, Gautam G. Rao, Jocelyn C. Reader, Pei Hui, Joan R. Tymon-Rosario, Justin Harold, Dennis Mauricio, Burak Zeybek, Gulden Menderes, Gary Altwerger, Elena Ratner, Alessandro D. Santin

**Affiliations:** 1 Marlene and Stewart Greenebaum Comprehensive Cancer Center, University of Maryland School of Medicine, Baltimore, MD USA; 2 University of Arkansas for Medical Sciences, Little Rock, AR USA; 3 Smilow Comprehensive Cancer Center, Yale School of Medicine, New Haven, CT USA; 4 Division of Gynecologic Oncology, Mercy Clinic, St. Louis, MO USA

Correction to: *British Journal of Cancer* 10.1038/s41416-022-01717-6, published online 11 February 2022

The article “Randomised phase II trial of weekly ixabepilone ± biweekly bevacizumab for platinum-resistant or refractory ovarian/fallopian tube/primary peritoneal cancer”, written by Dana M. Roque, Eric R. Siegel, Natalia Buza, Stefania Bellone, Dan-Arin Silasi, Gloria S. Huang, Vaagn Andikyan, Mitchell Clark, Masoud Azodi, Peter E. Schwartz, Gautam G. Rao, Jocelyn C. Reader, Pei Hui, Joan R. Tymon-Rosario, Justin Harold, Dennis Mauricio, Burak Zeybek, Gulden Menderes, Gary Altwerger, Elena Ratnerand and Alessandro D. Santin, was originally published electronically on the publisher’s internet portal on 11 February 2022 without open access. With the author(s)’ decision to opt for Open Choice the copyright of the article changed on 21 January 2024 to © The Authors 2024 and the article is forthwith distributed under a Creative Commons Attribution 4.0 International License, which permits use, sharing, adaptation, distribution and reproduction in any medium or format, as long as you give appropriate credit to the original author(s) and the source, provide a link to the Creative Commons licence, and indicate if changes were made. The images or other third party material in this article are included in the article’s Creative Commons licence, unless indicated otherwise in a credit line to the material. If material is not included in the article’s Creative Commons licence and your intended use is not permitted by statutory regulation or exceeds the permitted use, you will need to obtain permission directly from the copyright holder. To view a copy of this licence, visit http://creativecommons.org/licenses/by/4.0/

The original article has been corrected.

